# ^19^F Magnetic Resonance Imaging of Perfluorocarbons for the Evaluation of Response to Antibiotic Therapy in a *Staphylococcus aureus* Infection Model

**DOI:** 10.1371/journal.pone.0064440

**Published:** 2013-05-28

**Authors:** Tobias Hertlein, Volker Sturm, Peter Jakob, Knut Ohlsen

**Affiliations:** 1 Institute for Molecular Infection Biology, University of Würzburg, Würzburg, Germany; 2 Department of Experimental Physics 5, University of Würzburg, Würzburg, Germany; University Hospital Münster, Germany

## Abstract

**Background:**

The emergence of antibiotic resistant bacteria in recent decades has highlighted the importance of developing new drugs to treat infections. However, in addition to the design of new drugs, the development of accurate preclinical testing methods is essential. In vivo imaging technologies such as bioluminescence imaging (BLI) or magnetic resonance imaging (MRI) are promising approaches. In a previous study, we showed the effectiveness of ^19^F MRI using perfluorocarbon (PFC) emulsions for detecting the site of *Staphylococcus aureus* infection. In the present follow-up study, we investigated the use of this method for in vivo visualization of the effects of antibiotic therapy.

**Methods/Principal findings:**

Mice were infected with *S. aureus* Xen29 and treated with 0.9% NaCl solution, vancomycin or linezolid. Mock treatment led to the highest bioluminescence values during infection followed by vancomycin treatment. Counting the number of colony-forming units (cfu) at 7 days post-infection (p.i.) showed the highest bacterial burden for the mock group and the lowest for the linezolid group. Administration of PFCs at day 2 p.i. led to the accumulation of ^19^F at the rim of the abscess in all mice (in the shape of a hollow sphere), and antibiotic treatment decreased the ^19^F signal intensity and volume. Linezolid showed the strongest effect. The BLI, cfu, and MRI results were comparable.

**Conclusions:**

^19^F-MRI with PFCs is an effective non-invasive method for assessing the effects of antibiotic therapy in vivo. This method does not depend on pathogen specific markers and can therefore be used to estimate the efficacy of antibacterial therapy against a broad range of clinically relevant pathogens, and to localize sites of infection.

## Introduction

The emergence and spread of antibiotic resistant pathogens such as methicillin-resistant *Staphylococcus aureus* (MRSA) has led to a dramatic increase in the number of cases of invasive, life-threatening infection and a high rate of treatment failure in recent decades [1], [2]. Furthermore, MRSA strains were limited to hospital or clinical settings (hospital-acquired MRSA = ha-MRSA); however, they are now widespread in the community (community-associated MRSA = ca-MRSA) [3] and have even affected livestock (livestock-associated MRSA = la-MRSA) [4]. The multi-resistance pattern of MRSA strains restricts treatment options to a small subset of available antibiotics, and treatment with these antibiotics might lead to the emergence of resistant strains, resulting in an even more pronounced limitation of treatment options [5]. Therefore, it is of paramount importance to develop new promising drug candidates and lead compounds to combat bacterial infections in the future. In addition, the establishment of highly predictive in vivo models could help accelerate the selection process and capture the whole spectrum of activity of novel compounds in vivo. A promising approach to facilitate the screening process is the application of in vivo imaging technologies to preclinical testing [6], [7]. Technologies such as positron emission tomography, computed tomography, magnetic resonance (MRI) or bioluminescence imaging (BLI) enable the non-invasive visualization and quantification of disease progression in a longitudinal fashion [8]. These techniques may help limit the need for animal testing, in addition to providing detailed information about disease severity and complexity.

BLI is an effective method of visualizing the bacterial burden during an infection and has provided detailed information about the activity of antibiotics in several infectious disease models [9], [10]. However, one disadvantage of this technique is that it requires the introduction of a light-emitting luciferase into the pathogen of interest. This circumstance prohibits testing of new compounds against not genetically modified strains and cannot be easily transferred to the clinic, because pathogenic bacteria lack luciferase. On the other hand, MRI does not require a gene-reporter or a fluorescent molecule to visualize differences between infected and not-infected tissues. The use of MRI to assess the effects of antibacterial therapy was examined in a *S. aureus* thigh abscess model, in which changes in T_2_ relaxation times were compared with those observed in a mock-treated group; however, quantification and distinguishing between infected and not-infected tissues was difficult [11].

^19^F MRI using perfluorocarbon (PFC) emulsions might overcome this problem by enabling background-free visualization of the administered contrast media using objective measurement parameters [12], [13]. Intravenous administration of a PFC emulsion results in the accumulation of PFCs at the site of inflammation because they are phagocytosed by immune cells in the blood, which then migrate to inflammatory sites [14], [15]. We recently used a *S. aureus* thigh infection model to show that ^19^F MRI with PFCs enables visualization of the site of infection. The PFC tracer accumulates at the rim of the abscess in the shape of a hollow sphere, thereby defining the infected area [16].

Therefore, the aim of the present study was to determine the efficacy of ^19^F MRI for the non-invasive in vivo assessment of the effects of antibacterial therapy. Mice were infected with *S. aureus* and treated with two standard anti-MRSA antibiotics: vancomycin and linezolid. Comparative analysis of the ^19^F accumulation pattern, the bioluminescence signals generated by luciferase-expressing *S. aureus,* and the number of colony-forming units (cfu) showed that ^19^F-MRI with PFCs is an effective method for visualizing and quantifying the effects of antibiotic therapy in vivo.

## Results

### Bioluminescence Imaging of the Response to Antibacterial Therapy

**Figure 1:**
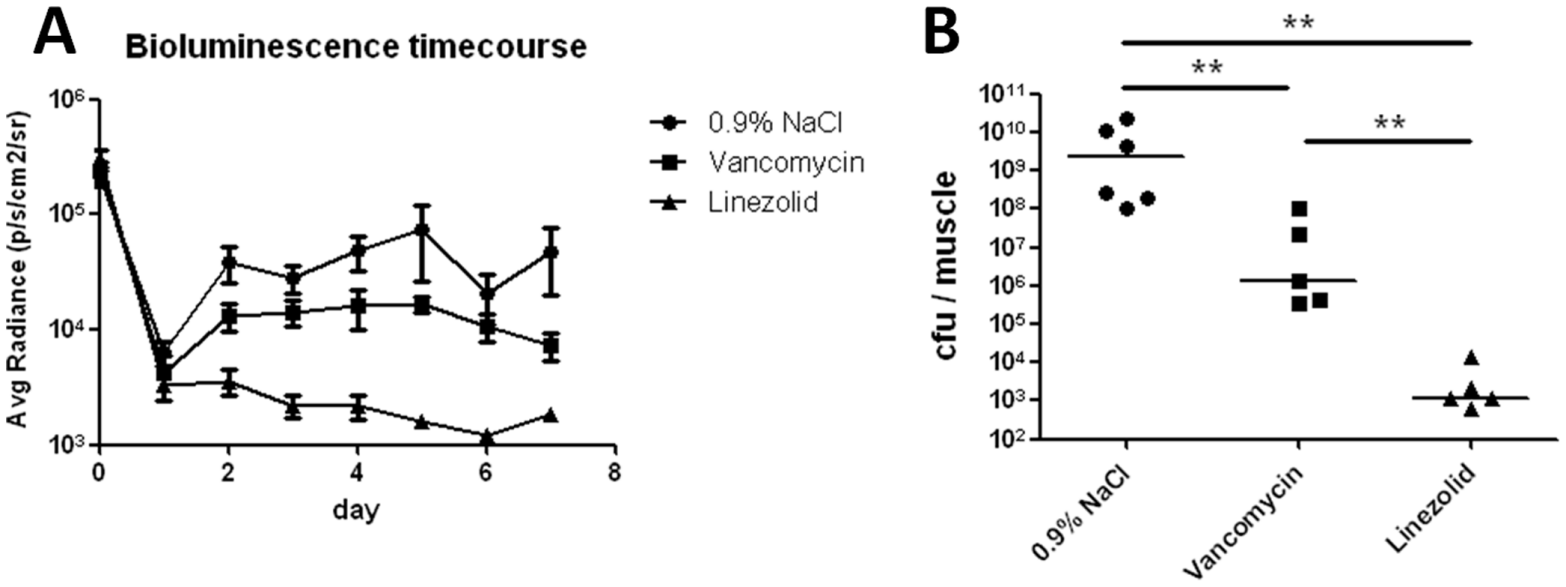
Effect of antibiotic therapy on bacterial burden in a thigh muscle abscess model. Bioluminescent *S. aureus* Xen29 (3.2×10^8^ cfu) was injected into the left thigh muscle of mice, which were treated with either 0.9% NaCl (n = 6), vancomycin (n = 5), or linezolid (n = 5). A) Luciferase expression in mice injected with *S. aureus* Xen 29 resulted in the emission of bioluminescence at the site of infection. Mean (± SEM) bioluminescence values are shown at the indicated time points. B) Infected thigh muscles were harvested on day 7 post-infection and the number of colony-forming units was counted after homogenization and plating. The cfu values for each infected muscle, and the median number for each group, are shown. Statistically significant differences between the groups are indicated by asterisks (**P<0.01).

The *S. aureus* strain, Xen29, which harbors a stable copy of the *Photorhabdus luminescens luxABCDE* operon and therefore emits a bioluminescent signal [9], was injected (at a dose of 3.2×10^8^ cfu) into the left thigh muscle after a first control BL image was obtained. The average radiance was quantified applying a standardized oval area of interest to all measurements (Fig. 1A). High photon emission was observed 15 min after the injection of bacteria, but the signal decreased sharply until day 1 post-injection (p.i.) in all groups. In the mock-treated group, the average radiance increased and reached a plateau phase between day 3 and day 7 p.i. Treatment with either vancomycin or linezolid resulted in a reduction in bioluminescence (beginning on day 2 p.i.), compared with that in the mock-treated group. The greatest reduction was observed in response to linezolid treatment. The mean average radiance on day 3 p.i. was 2.8±0.76×10^4^ p/s/cm^2^/sr for the mock-treated group, 1.4±0.36×10^4^ p/s/cm^2^/sr for the vancomycin group, and 2.19±0.48×10^3^ p/s/cm^2^/sr for the linezolid group (p<0.01 *vs.* 0.9% NaCl; p<0.05 *vs.* vancomycin). On day 7 p.i., the mean average radiance values at the area of interest for the mock, vancomycin, and linezolid groups were 4.79±2.76×10^4^, 7.40±2.02×10^3^ (p<0.05 *vs.* 0.9% NaCl group) and 1.85±0.15×10^3^ p/s/cm^2^/sr (p<0.01 *vs*. 0.9% NaCl; p<0.05 *vs.* vancomycin), respectively. These results indicate that, when compared with the 0.9% NaCl-treated group, vancomycin treatment reduced the bioluminescent signal by 49% on day 3 p.i., and by 85% on day 7 p.i. Linezolid led to a 92% reduction on day 3 p.i. and a 96% reduction on day 7 p.i.

### Bacterial Burden on Day 7 Post-infection

The muscles were harvested on day 7 p.i., homogenized, and the number of colony-forming units was determined (Fig. 1B). The mean cfu values in the infected thigh muscle were 6.55±3.74×10^9^ for the mock-treated group, 2.5±1.97×10^7^ for the vancomycin-treated group (2 log10 units lower than that in the mock control) and 3.8±2.58×10^3^ for the linezolid-treated group (a 6 log10 unit reduction).

### Visualization of ^19^F at the Site of Infection during Antibiotic Therapy

**Figure 2:**
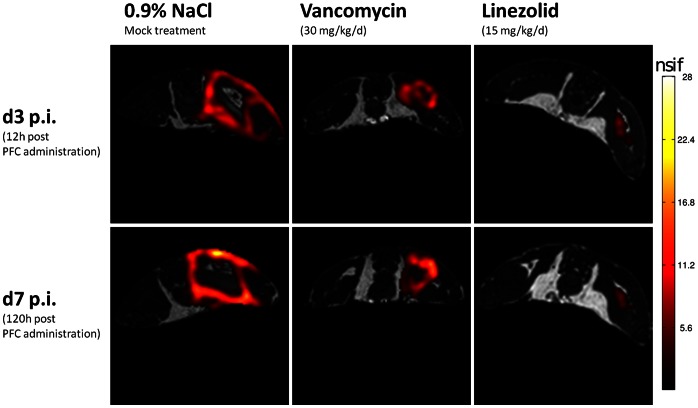
Representative 19F chemical shift imaging steady-state free precession overlays on 1H-turbo spin echo scans of one mouse from each group during infection and antibiotic therapy. *S. aureus* Xen 29 (3.2×10^8^) was injected into the left thigh muscle and the PFC tracer was administered intravenously 48 h post-infection. Each mouse underwent magnetic resonance imaging at 12 h and 120 h after PFC administration. All groups showed strong accumulation of ^19^F at the rim of the abscess area. The ^19^F signal intensity is indicated by the hot (red to white) color scale superimposed over a grey ^1^H image, which shows the anatomical context.

Circulating phagocytic cells incorporate intravenously-administered PFC emulsions and migrate to the site of inflammation, leading to the accumulation of the tracer at this site [14], [17]. In the *S. aureus* thigh abscess model, administration of PFC to the mock-treated group resulted in the accumulation of the ^19^F tracer at the rim of the abscess, which was consistent with the results of our previous study [16]. Vancomycin treatment reduced the size of the abscess, and linezolid had an even greater effect (Fig. 2). Nevertheless, the pattern of ^19^F accumulation at the rim of the abscess was, in terms of geometry, comparable in all three groups.

### Estimation of ^19^F Accumulation at the Site of Infection during Antibiotic Therapy

**Figure 3:**
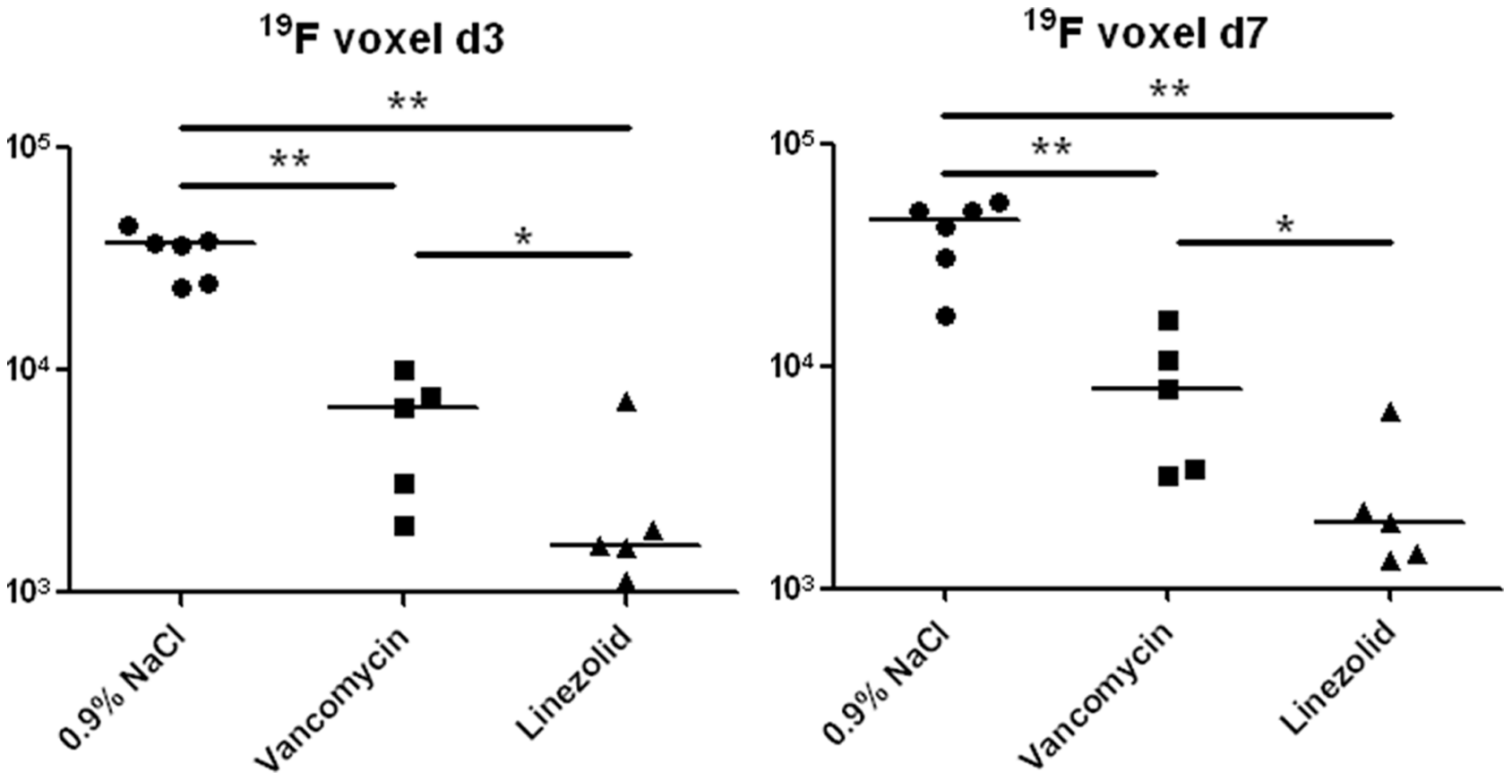
19F filled volume in the infected thigh muscle at days 3 and 7 post-infection. *S. aureus* Xen 29 (3.2×10^8^) was injected into the left thigh muscle and the PFC tracer was administered intravenously 48 h post-infection. Mice underwent MRI at 12 h and 120 h after PFC administration. The number of ^19^F filled voxels in each infected muscle and the median for each group are shown. Statistically significant differences between the groups are indicated by asterisks (*P<0.05, **P<0.01).

To estimate the volume of the abscess, we measured the accumulation of the ^19^F tracer at the site of infection and calculated the total volume of ^19^F-accumulation by adding all ‘fluorine filled’ voxels. The corresponding values are plotted in Fig. 3. The highest ^19^F volumes were detected in the mock-treated group on days 3 and 7 p.i. (3.43±0.34×10^4^ and 4.06±0.58×10^4^ voxels, respectively). The values in the vancomycin-treated group were 5.94±1.48×10^3^ and 8.25±2.40×10^3^ voxels at days 3 and 7 p.i., respectively (p<0.01 *vs.* 0.9% NaCl at both time points). The ^19^F volumes in the linezolid-treated group were 2.73±1.17×10^3^ and 2.66±0.93×10^3^ voxels at days 3 and 7 p.i., respectively (p<0.01 *vs.* 0.9% NaCl and p<0.05 *vs.* vancomycin at both time points). These results indicate that, compared with the mock-treated group, vancomycin reduced the ^19^F volume by 83% and 80%, and linezolid reduced the ^19^F volume by 92% and 93% at days 3 and 7 p.i., respectively.

**Figure 4:**
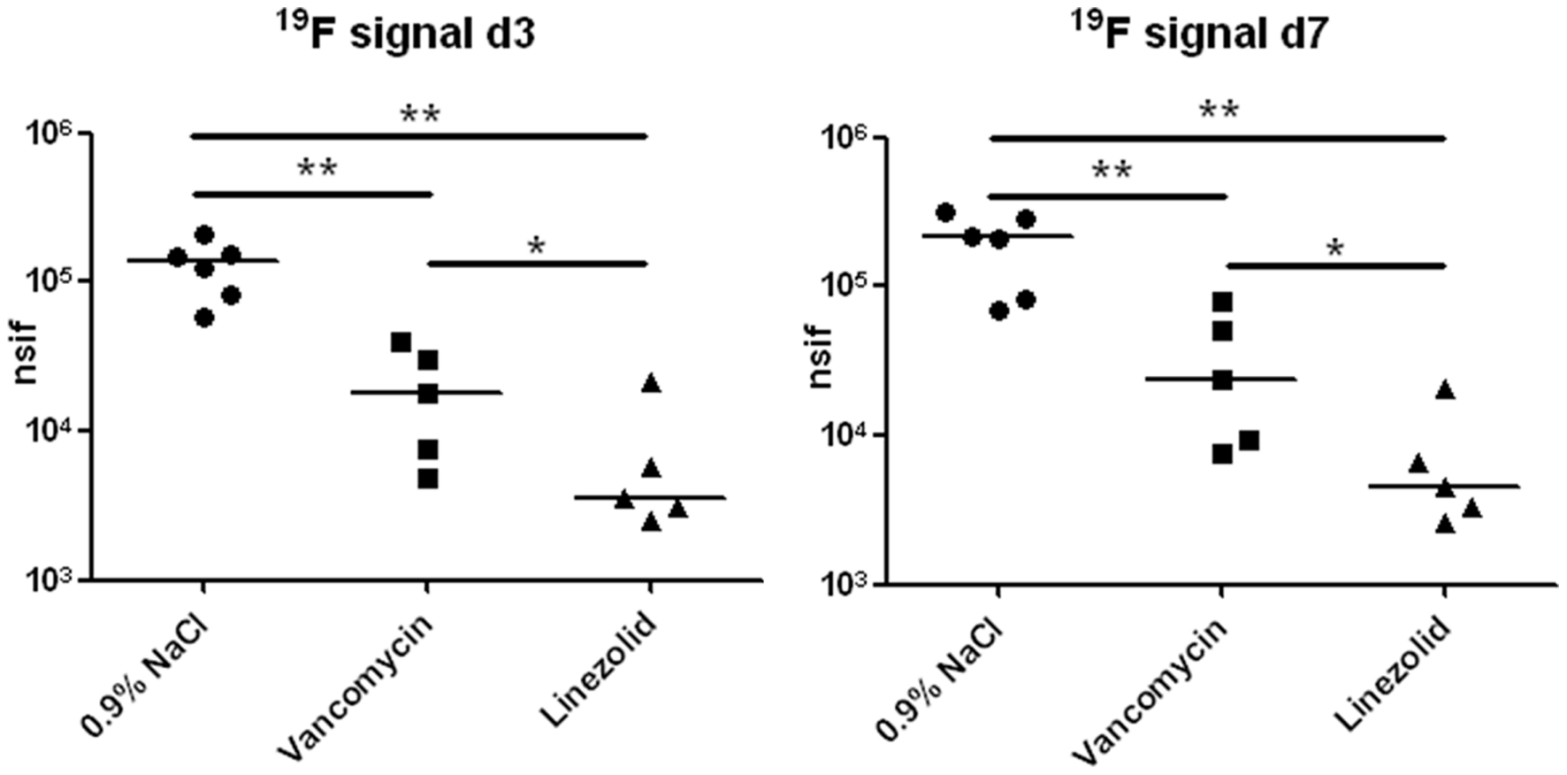
Overall 19F signal in the infected thigh muscle at days 3 and 7 post-infection. *S. aureus* Xen 29 (3.2×10^8^) was injected into the left thigh muscle and the PFC tracer was administered intravenously 48 h post-infection. Mice underwent MRI at 12 h and 120 h after PFC administration. The overall ^19^F signal of all ^19^F filled voxels in each infected thigh muscle and the median for each group are shown. Statistically significant differences between the groups are indicated by asterisks (*P<0.05, **P<0.01).

Because phagocytosis of the ^19^F tracer by immune cells leads to its accumulation at the site of inflammation, the total ^19^F signal in the abscess area can serve as a surrogate marker for the immune response [14], [17]. Therefore, we calculated the total ^19^F signal by adding the signals of all individual ^19^F pixels over the entire abscess area (Fig. 4). The value for the mock-treated group was 1.29±0.22×10^5^ nsif (^19^F MR signal intensity normalized to the apparent noise amplitude of the measurement) at day 3 and 1.97±0.41×10^5^ nsif at day 7 p.i. In the vancomycin-treated group, these values were 2.03±0.67×10^4^ and 3.41±1.37×10^4^ nsif at days 3 and 7 p.i., respectively (p<0.01 *vs.* 0.9% NaCl at both time points). The values for linezolid were 7.31±3.64×10^3^ and 1.21±0.46×10^4^ nsif at days 3 and 7 p.i., respectively (p<0.01 *vs.* 0.9% NaCl and p<0.05 *vs.* vancomycin at both time points). These results show that the highest values for the ^19^F signal were detected in the mock-treated group, and that vancomycin treatment reduced the signal intensity by 84% and 83% at days 3 and 7 p.i., respectively, whereas the reduction induced by linezolid was 94% and 96% at days 3 and 7 p.i., respectively.

## Discussion

The present study investigated the use of ^19^F MRI using PFCs as a tracer to visualize and evaluate responses to antibiotic therapy in a preclinical infectious disease model. A non-invasive imaging method for the in vivo screening of new drugs may provide accurate and detailed information about disease progression and minimize the use of experimental animals [6]. The recent development of BLI has enabled the imaging of bacterial infection and the visualization and quantification of the effects of antibacterial therapy in vivo [9], [10]. Therefore, the present study used BLI as a reference method for determining the efficacy of ^19^F MRI using PFCs as platform for assessing the effects of antibiotics.

In our experimental model, animals were infected with *S. aureus* Xen29, which was derived from strain NCTC8532 via the insertion of the *luxABCDE* operon at a single chromosomal site [9], to enable BLI during antibiotic treatment. The advantage of in vivo BLI is that the same animal can be imaged at several time points during the course of infection, thereby minimizing individual variations in infected animals. Untreated control animals showed the highest levels of photon emission, with vancomycin or linezolid treatment leading to a reduction in the bioluminescence signal during the course of infection. Quantification of the bacterial burden at day 7 p.i. showed that treatment with vancomycin resulted in a 100-fold reduction in *S. aureus*; however, linezolid had a greater effect, reducing the bacterial burden by 6 log10 units compared with that in the mock-treated control group. These results are in line with those of previous studies showing that vancomycin has relatively weak antibacterial activity in murine abscess models [11], [18]. Quantification of both cfu and BL signal intensity showed the efficacy of both antibiotics, and that linezolid was superior to vancomycin in this model; however, the cfu data revealed a greater difference between the two antibiotics.

These methods assess bacterial burden either directly (cfu counting) or indirectly (BLI). Therefore, we tried to determine whether ^19^F MRI using PFCs allows the visualization of the infection process and the in vivo assessment of the activity of antibiotics in a non-invasive manner. In this regard, ^19^F MRI with PFCs cannot directly determine bacterial numbers during the infection process; rather, it enables the visualization of the host immune response by labeling phagocytic immune cells [14], [17]. Our main goal was to identify a method capable of providing information about the infection process in vivo using non-invasive imaging technology.

In a previous study, we showed that intravenous administration of PFCs as early as 48 h p.i. led to a stable ^19^F signal at the site of infection up until at least 9 days p.i. [16]. However, the ^19^F accumulation pattern at the site of infection, and the response to antibiotic therapy, has not been examined to date. In all animals, including those treated with antibiotics, ^19^F accumulated at the rim of the abscess, forming a hollow sphere. These results indicated that antibiotic therapy did not prevent PFC accumulation at the site of infection, nor did it inhibit visualization of the abscess area by ^19^F MRI. Since the three dimensional accumulation pattern did not form a completely closed hollow sphere the volume of the abscess area could not be measured directly (see S1–S6). Therefore, we estimated the infection volume indirectly by measuring the accumulation of PFC-filled voxels around the infection area. The calculated volume of PFC-filled voxels correlated qualitatively with treatment efficacy, as indicated by bioluminescence imaging and cfu data. The second quantifiable parameter, the overall ^19^F signal strength in the infected thigh muscle, also showed the highest value in the mock-treated group and the lowest in the linezolid group, with an intermediate value in response to vancomycin (Fig. 4); these results were in agreement with the results of bioluminescence imaging and cfu determination. Bioluminescence accumulation and ^19^F filled volume or signal intensity identified comparable differences in magnitude between the treatment groups, whereas cfu determination detected greater differences. These results suggest that the number of phagocytic cells in an abscess area correlates with the number of live *S. aureus* bacteria within the abscess. A small increase in the ^19^F signal was observed between day 3 p.i. (12 h after PFC administration) and day 7 p.i. in all treatment groups, indicating that the PFC tracer was transported to the site of infection more than 12 h post-administration, and was retained there for a long time.

Overall, both the ^19^F filled volume and the ^19^F signal intensity in the infected thigh muscle correlated with the efficacy of the tested antibiotics at both time points, with the highest signals observed in the mock-treated group and the lowest in the linezolid-treated group. ^19^F MRI with PFCs is independent of the infecting pathogen, because the contrast media accumulates at the site of inflammation after phagocytosis by circulating immune cells and their subsequent migration [14], [15]. Therefore, this method may be useful for assessing inflammatory responses to new drug candidates by measuring the number of phagocytic cells at the site of infection. Furthermore, ^19^F MRI delivers a more detailed picture of the state of disease than the classical determination of bacterial burden because it allows the visualization of the tissue at the site of infection, pathogen induced changes, and the quantification of phagocytes. Further studies are necessary to assess the potential of ^19^F MRI to detect infection foci of unknown origin. In medical practice, severe and complicated infections caused by *S. aureus,* such as osteomyelitis, are often difficult to recognize and are associated with high rates of treatment failure. Therefore, accurate localization of the infection focus could help determine the most appropriate therapeutic measures. Moreover, the visualization and assessment of soft tissue abscesses could be a promising approach to tracking and measuring the efficacy of antibiotic therapy.

In conclusion, the results of the present study indicate that the accumulation of PFC emulsions at the site of infection reflects the immune response to antibacterial therapy, and is therefore a useful approach to evaluating antibiotics in preclinical infectious disease models. Visualization of PFC accumulation by ^19^F MRI might enable the clinician to differentiate between infected and non-infected tissues, and is a promising tool for indirectly estimating the abscess area and the effects of antibiotics.

## Materials and Methods

### Ethics Statement

The present study was approved by the Committee on the Ethics of Animal Experiments of the government of Lower Franconia (55.2-2531.01-06/12) and all experiments were performed in strict accordance with the German animal protection laws. All animals were kept in cages under standardized lighting conditions and had access to food and water ad libitum. In vivo imaging was performed under isoflurane anesthesia, and all efforts were made to minimize suffering. Animals were sacrificed at the end of the experiment by CO_2_ inhalation.

### Materials

Vancomycin and linezolid were purchased from Sigma Aldrich (St. Louis, Mo, USA) and dissolved in sterile 0.9% NaCl solution. The ^19^F nanoemulsion core compound was PFC, with a mean droplet diameter of approximately 145 nm. A 20% (v/v) emulsion (VS1000H, Celsense, Inc., Pittsburgh, USA) was used for direct intravenous injection.

### Murine Thigh Abscess Model

The murine *S. aureus* thigh infection model was selected because of the localized and bulky abscesses generated by this model [19]. A total of 16 female Balb/C mice (18–22 g, Charles-River, Germany) were used for the study. The animals were anesthetized with isoflurane (1.5% isoflurane in O_2_) and 50 µL of bacterial suspension (3.2×10^8^ cfu per infection dose) in 0.9% NaCl was inoculated into the left thigh muscle. The bacterial suspension was prepared by overnight incubation of *S. aureus* Xen 29 (Caliper Life Sciences, Waltham, MA, USA) in liquid B-medium. The bacteria were then washed, resuspended in 0.9% NaCl and diluted to a final concentration of 6.4×10^9^ cfu/mL. The infection dose was controlled by optical density and assessed by plating dilutions on agar plates.

The mice were then assigned to one of three treatment groups. The mock-treated group (0.9% NaCl group) received 50 µL of 0.9% NaCl i.p. 2 h p.i, and then every 12 h thereafter. The antibiotic treatment groups (vancomycin or linezolid) received the first antibiotic treatment 2 h p.i. and then every 12 h thereafter. The dosing regimen was 30 mg/kg/d for vancomycin and 15 mg/kg/d for linezolid. All mice received a single intravenous dose (100 µL) of PFC contrast media on day 2 p.i. Photon emission was detected by BLI before, at 15 min p.i., and then every 24 h. The accumulation of ^19^F at the site of infection was visualized by MRI at day 3 p.i. (12 h post-PFC administration) and at day 7 p.i. (5 days post-PFC administration). The animals were sacrificed after the final MRI on day 7 p.i. by inhalation of pure CO_2_.

### Magnetic Resonance Imaging

MRI experiments were performed on a 7 Tesla Bruker Biospec System (Bruker BioSpin GmbH, Reinstetten, Germany) at room temperature with a double resonant (^1^H and ^19^F) home-built birdcage. The animals were anesthetized (1% isoflurane) and placed in a home-built container to comply with safety regulations.

^1^H MRI anatomical imaging was performed by a turbo spin echo (TSE) sequence (TEeff/TR: 20.4 ms/500 ms; inter-echo time: 5.1 ms; turbo factor: 4; FOV: 25×25×25 mm^3^; matrix: 100×96×100; NA: 1).

For ^19^F-MRI, two acquisition-weighted [20] 3D steady-state free precession chemical shift imaging (SSFP-CSI) sequences [21] were performed with the same geometry as that used for the ^1^H-TSE scans, but with different pulse phase increments (θ: 0°/180°, pulse shape: hermite; pulse bandwidth: 5400 Hz; TACQ/TR: 10.24 ms/13.1 ms; FOV: 25×25×16 mm^3^; spectral points: 512; acquired resolution/time equivalent: - matrix: 31×31×20; - NA: 4). The overall protocol took less than 1.5 h. The two acquired datasets were combined using “sum-of-squares” (SoS) to reduce the banding artefacts. The ^19^F spectral line was pixel-wise summed up and the resulting data were zero-filled to a matrix of 100×100×64 pixels.

### MR-Quantification

Because of time restrictions for the in vivo experiments, ^19^F B_1_ maps, which are mandatory for absolute MR signal quantification, could not be acquired for every mouse. Therefore, absolute quantification of the ^19^F content was not possible. To minimize this B_1_-effect and achieve the highest comparability between all experiments, we used mice of matching size and weight that were similarly positioned in the rather homogeneous B_1_-field of a birdcage coil. Consequently, we used the ^19^F MR signal intensity, normalized to the apparent noise amplitude of the measurement (nsif), as an estimate of the ^19^F content. The apparent mean noise amplitude and the variation of the background signal σ of the processed ^19^F images was thereby determined by fitting the histogram of the pixel intensities, using a Gaussian noise model as an approximation. To avoid false positive events, we considered a voxel as ‘fluorine filled’ if the corresponding ^19^F signal intensity was greater than the apparent mean noise amplitude plus 4 σ. This threshold was used for all further examinations.

### Bioluminescence Imaging

BLI was performed with an In Vivo Imaging System (IVIS Lumina II, Caliper Life Sciences, USA). The mice were anesthetized with 2% isoflurane and imaged using the following parameters: exposure, 120 s; FStop, 1; excitation, block; emission, open; FOV, D; height, 1.5 cm. A standardized area of interest was manually defined at the site of infection, with the same size and geometry for all mice and all time points. This was necessary to determine the values for all animals because of the lack of signal above background noise in some mice, in particular those in the linezolid group. To overcome this, we defined an area of interest at the site of infection to determine average radiance values for comparison between groups. The average radiance values were then analyzed using Living Image 3.2 software (Caliper Life Sciences, USA).

### Counting the Number of Colony-forming Units

The infected thigh muscles were removed on day 7 p.i. and homogenized in a tissue homogenizer. The homogenate was then serially diluted in sterile 0.9% NaCl, and several dilutions were plated on B agar plates and cultivated overnight. The number of colonies on the plates was then counted.

### Statistical Analysis

Data analysis was performed using GraphPad Prism 5.0 software (GraphPad, San Diego, USA) and groups were compared using the Mann-Whitney U test (*P<0.05, **P<0.01). The results are expressed as the median value of all measurements and groups (with the exception of Fig. 1, in which the mean BL signal ± SEM is shown).

## Supporting Information

Video S1 Representative 3D visualization of 19F chemical shift imaging steady-state free precession overlays on 1H- turbo spin echo scans at day 3 post-infection in mock-treated mice. *S. aureus* Xen 29 was injected into the left thigh muscle of the mice and a perfluorocarbon (PFC) emulsion was administered intravenously 48 h later. MRI was performed 24 h after PFC administration. The video was generated using MeVislab (Fraunhofer Mevis, Bremen, Germany).
(AVI)

Video S2 Representative 3D visualization of 19F chemical shift imaging steady-state free precession overlays on 1H- turbo spin echo scans at day 7 post-infection in mock-treated mice. *S. aureus* Xen 29 was injected into the left thigh muscle of the mice and a perfluorocarbon emulsion was administered intravenously 48 h later. MRI was performed on day 7 p.i. The video was generated using MeVislab (Fraunhofer Mevis, Bremen, Germany).
(AVI)

Video S3 Representative 3D visualization of 19F chemical shift imaging steady-state free precession overlays on 1H- turbo spin echo scans at day 3 post-infection during Vancomycin treatment. *S. aureus* Xen 29 was injected into the left thigh muscle of the mice and a perfluorocarbon (PFC) emulsion was administered intravenously 48 h later. Vancomycin (30 mg/kg/d) was administered intraperitoneally 2 h post-infection, and then every 12 h thereafter. MRI was performed 24 h after PFC administration. The video was generated using MeVislab (Fraunhofer Mevis, Bremen, Germany).
(AVI)

Video S4 Representative 3D visualization of 19F chemical shift imaging steady-state free precession overlays on 1H- turbo spin echo scans at day 7 post-infection during Vancomycin treatment. *S. aureus* Xen 29 was injected into the left thigh muscle of the mice and a perfluorocarbon emulsion was administered intravenously 48 h later. Vancomycin (30 mg/kg/d) was administered intraperitoneally 2 h post-infection and then every 12 h thereafter. MRI was performed on day 7 post-infection. The video was generated using MeVislab (Fraunhofer Mevis, Bremen, Germany).
(AVI)

Video S5 Representative 3D visualization of 19F chemical shift imaging steady-state free precession overlays on 1H- turbo spin echo scans at day 3 post infection during Linezolid treatment. *S. aureus* Xen 29 was injected into the left thigh muscle of the mice and a perfluorocarbon (PFC) emulsion was administered intravenously 48 h later. Linezolid (15 mg/kg/d) was administered intraperitoneally 2 h post-infection and then every 12 h thereafter. MRI was performed 24 h after PFC administration. The video was generated using MeVislab (Fraunhofer Mevis, Bremen, Germany).
(AVI)

Video S6 Representative 3D visualization of 19F chemical shift imaging steady-state free precession overlays on 1H- turbo spin echo scans at day 7 post infection during Linezolid treatment. *S. aureus* Xen 29 was injected into the left thigh muscle of the mice and a perfluorocarbon emulsion was administered intravenously 48 h later. Linezolid (15 mg/kg/d) was administered intraperitoneally 2 h post-infection and then every 12 h thereafter. MRI was performed at day 7 post-infection. The video was generated using MeVislab (Fraunhofer Mevis, Bremen, Germany).
(AVI)
